# Treatment of myocardial infarction via sodium alginate microsphere-mediated co-delivery of *Corydalis yanhusuo* ethanolic extract and mesenchymal stem cells

**DOI:** 10.1186/s13020-026-01557-z

**Published:** 2026-10-30

**Authors:** Yuanye Dang, Junjie Cai, Yongxian Li, Xudong Qin, Jun Shen, Lian Duan, Qingyun Lu, Xinyue Zhang, Wenqi Xiao, Ruoxuan Liu, Guosheng Tang, Ailan Chen, Huaibin Wang

**Affiliations:** 1 Guangzhou Municipal and Guangdong Provincial Key Laboratory of Molecular Target and Clinical Pharmacology, The NMPA and State Key Laboratory of Respiratory Disease, School of Pharmaceutical Sciences, Guangzhou Medical University, Guangzhou, 511436 China; 2 Department of Cardiology, The First Affiliated Hospital of Guangzhou Medical University, China State Key Laboratory for Respiratory Disease, Guangzhou, 510120 China; 3 Phase I Clinical Trial Center, The Second Affiliated Hospital, Guangzhou Medical University, Guangzhou, 511400 China; 4 Department of Pharmacy, The Affiliated Traditional Chinese Medicine Hospital, Guangzhou Medical University, Guangzhou, 510645 China

**Keywords:** Myocardial infarction, *Corydalis yanhusuo* ethanolic extract, Stem cell therapy, Sodium alginate microspheres, Synergistic therapy, Inflammation

## Abstract

Persistent inflammatory responses and adverse ventricular remodeling following Acute Myocardial Infarction (MI) are critical factors driving the progressive decline of cardiac function. Traditional pharmacological strategies struggle to achieve effective long-term intervention at the level of tissue repair. The *Corydalis yanhusuo* ethanolic extract (CYEE) possesses demonstrated anti-inflammatory and antioxidant activities; however, its limited in vivo half-life and the difficulty in maintaining local effective concentrations hinder its efficacy. Concurrently, while mesenchymal stem cells (MSCs) exert therapeutic potential through paracrine and immunomodulatory mechanisms, their low viability within the intensely inflammatory microenvironment of the infarcted myocardium significantly restricts their clinical impact. In this study, a sodium alginate microsphere delivery system was developed using gas-assisted microfluidic technology to achieve the synergistic encapsulation and localized delivery of CYEE and MSCs. This oil-free and surfactant-free microsphere system exhibits excellent biocompatibility, enabling the sustained release of CYEE within the infarcted region to alleviate inflammation and oxidative stress for initial therapeutic intervention. Furthermore, it enhances MSCs viability and paracrine function by optimizing the local microenvironment. In vitro experiments demonstrated that the system significantly improved cell viability under inflammatory conditions and promoted migration and angiogenic capacity. In a rat MI model, synergistic delivery significantly improved cardiac function and attenuated myocardial fibrosis and adverse remodeling without evident systemic toxicity. These results indicate that combining CYEE-mediated microenvironmental regulation with the dynamic synergistic repair of MSCs provides a comprehensive therapeutic strategy with potential translational value for myocardial repair following infarction.

**Graphical Abstract:**

**Figure:**
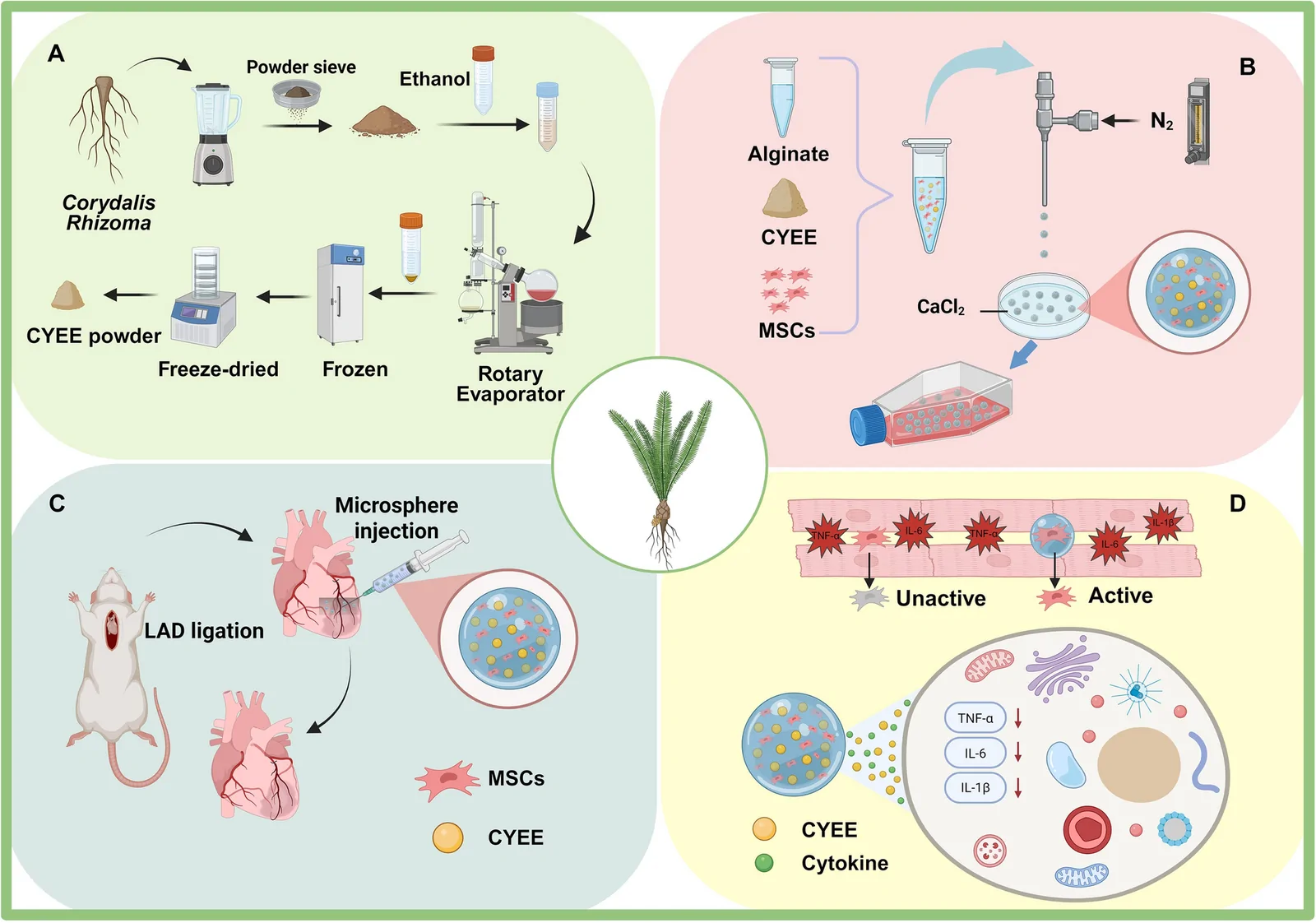

**Supplementary Information:**

The online version contains supplementary material available at https://doi.org/10.1186/s13020-026-01557-z.

## Introduction

Coronary artery disease (CAD) is a primary etiology of heart failure. As the terminal stage of most ischemic heart diseases, heart failure continues to exhibit rising morbidity and mortality rates [1, 2]. Acute myocardial infarction (MI) represents the most severe clinical manifestation of CAD. Its occurrence not only results in the irreversible necrosis of a vast number of cardiomyocytes, but also triggers persistent inflammatory responses and adverse ventricular remodeling, ultimately leading to progressive decline in cardiac function [3, 4].

Currently, clinical treatment relies predominantly on revascularization strategies, such as percutaneous coronary intervention (PCI) and coronary artery bypass grafting (CABG), to restore blood perfusion to the ischemic myocardium as early as possible [5, 6]. These surgical interventions are of irreplaceable value in reducing acute-phase mortality, limiting the infarct area, and rapidly alleviating ischemia-related symptoms. However, these strategies primarily target the blood flow obstruction itself; they are unable to reverse established cardiomyocyte apoptosis and necrosis, nor can they effectively block the subsequent inflammatory cascade and pathological ventricular remodeling processes [7, 8]. Pharmacotherapy (e.g., thrombolytics, antiplatelet agents, and lipid-lowering drugs) remains foundational in the long-term management of myocardial infarction, yet its capacity for intervention at the level of tissue repair is limited [9, 10]. Conventional drugs typically target single or limited pathways and rely on passive diffusion for therapeutic effect, making it difficult to maintain stable, stage-appropriate effective concentrations within the infarcted region [11, 12]. Furthermore, the intensity and characteristics of the post-MI inflammatory response evolve dynamically over the course of the disease [13], making it challenging for single, short-acting drug intervention models to precisely regulate this complex biological process [14]. Therefore, exploring comprehensive therapeutic strategies capable of regulating inflammation, attenuating cardiomyocyte apoptosis, and promoting ischemic angiogenesis, thereby achieving multi-level and sustained microenvironmental remodeling, has become a vital direction in myocardial infarction research [15]. Such strategies hold the potential to overcome the limitations of single-target and short-acting interventions, providing a new theoretical foundation and technical pathway for the long-term repair of cardiac structure and function.

Traditional Chinese medicine (TCM) and its active extracts offer unique advantages in the prevention and treatment of cardiovascular diseases due to their multi-component, multi-target, and holistic regulatory characteristics [16]. Extracts from *Corydalis yanhusuo* (Papaveraceae) are rich in various alkaloids with definitive pharmacological activities [17, 18]. These have been demonstrated to possess significant anti-inflammatory, analgesic, antioxidant, and anti-tumor effects, as well as the ability to mitigate ischemic myocardial injury [19–22]. However, systematic research on its role and potential mechanisms during the cardiac repair process following myocardial infarction is still lacking. The persistent inflammatory microenvironment after MI is a core factor limiting cardiac repair. By improving the local tissue environment through anti-inflammatory regulation [22], *Corydalis yanhusuo* ethanolic extract (CYEE) helps maintain cardiomyocyte viability during ischemia and may play an important role in myocardial repair. Nevertheless, like many natural plant-derived bioactive alkaloids, CYEE suffers from limited in vivo half-life and the difficulty in maintaining local effective concentrations, which continue to restrict the full realization of its therapeutic potential [23, 24]. In recent years, cell therapy has provided new research perspectives for myocardial repair, with mesenchymal stem cells (MSCs) receiving extensive attention for their immunomodulatory and paracrine capabilities [25]. Existing studies indicate that the restorative effects of MSCs depend primarily on paracrine pathways, including the secretion of cytokines, chemokines, and exosomes, which play key roles in promoting angiogenesis, inhibiting inflammation, and regulating the fibrotic process [26]. However, the intense inflammatory response and oxidative stress environment within the infarcted myocardium significantly reduce MSCs viability. Simple cell injections often result in the rapid loss of transplanted cells, thereby weakening their paracrine function and dynamic regulatory capacity, which limits the efficacy of cell therapy [27]. Consequently, the synergistic co-delivery of drugs and cells has great potential to compensate for the shortcomings of monotherapies. This approach can provide initial treatment and improve the local microenvironment while creating stable, favorable conditions for MSCs survival and function, thereby achieving synergistically enhanced myocardial repair.

Hydrogels have been widely utilized as carriers for drug or cell delivery; however [28], the presence of bulk hydrogel nanonetworks often restricts cell viability and mass transfer efficiency [29]. Microgels are three-dimensional porous polymer networks characterized by excellent hydrophilicity and biocompatibility [30]. The implantation of injectable microgels into damaged areas can provide mechanical support for the myocardial wall, alleviating mechanical load and left ventricular wall stress, thereby preventing ventricular remodeling [31]. More importantly, microgels serve as optimal carriers for drug and cell delivery, ensuring the efficient and sustained release of therapeutic agents while providing physical protection for cells. This protection mitigates adverse effects such as inflammation and oxidative stress, ultimately promoting myocardial regeneration and repair [32]. Based on these advantages, we have developed a synergistic delivery system for CYEE and MSCs using sodium alginate (SA) microspheres, and systematically evaluated its therapeutic efficacy and underlying mechanisms in myocardial infarction repair. CYEE provides initial therapeutic intervention through anti-inflammatory and anti-oxidative stress activities, interrupting the post-infarction inflammatory cascade and improving the local pathological microenvironment at its source. As biological regulatory units highly sensitive to microenvironmental changes, MSCs exhibit enhanced viability and paracrine functions, which amplify the overall regenerative effect [33].

In this study, sodium alginate microspheres were fabricated using gas-shearing microfluidic technology. This technique requires no oil phase or surfactants; the preparation process is mild and highly controllable, significantly reducing potential damage to cellular viability and function. It represents a highly cell-friendly strategy for microsphere construction [34–36]. Its superior biocompatibility and mild ionic crosslinking properties enable the stable co-loading of drugs and cells without compromising biological activity [37]. The microsphere structure not only facilitates the local enrichment and sustained release of CYEE, extending its anti-inflammatory and anti-oxidative effects for initial therapy, but also provides physical protection for MSCs while optimizing the local microenvironment. Furthermore, the excellent injectability, tissue adaptability, and porous network of the microspheres allow encapsulated cells to fully exchange signaling molecules with the external environment. This enables a dynamic response to the improved microenvironment, further promoting synergistic repair (Graphical abstract). It is worth emphasising that the core innovation of this study is not limited to the simple combined delivery of drugs and cells, but lies in the development of a time-coordinated myocardial repair strategy based on the pre-regulation of the inflammatory microenvironment. The persistent inflammatory response following myocardial infarction not only directly exacerbates myocardial damage, but also constitutes a major obstacle to the survival, retention and functional performance of MSCs. We therefore propose a 'microenvironment-first' therapeutic approach, whereby the sustained release of CYEE is utilised to first alleviate local inflammation and oxidative stress, thereby reshaping the microenvironment to favour tissue repair. This subsequently promotes the long-term survival and paracrine function of MSCs in the infarct area, thereby achieving a continuous therapeutic process ranging from inflammation control to tissue regeneration.

This research is the first to experimentally confirm the direct role of CYEE in myocardial infarction repair and elucidates the potential mechanism by which it enhances MSCs-mediated synergistic repair through anti-inflammatory microenvironment regulation. These findings have provided a new theoretical basis for the modern translation of traditional Chinese medicine extracts in treating myocardial infarction and offered a novel approach for constructing multi-strategy synergistic myocardial repair systems combining pharmacological and cellular therapies.

## Materials and methods

### Materials

Sodium Alginate (Aladdin, S100128), Calcium Chloride (CaCl₂, Analytical Grade), Fluorescent Polystyrene Nanoparticles (Dae), Dulbecco’s Modified Eagle Medium (DMEM, Gibco), Minimum Essential Medium (MEM, Gibco), and Calcein/Propitium Iodide (Calcein/PI) Live/Dead Cell Staining Kit (C2015M, Beyotime), primary antibodies: cTnT (Servicebio, GB11364, 1:500), α-SMA (Servicebio, GB121364, 1:2500), Ki67 (Servicebio, GB111141, 1:2500), CD86 (Servicebio, GB150054, 1:500) and CD206 (Servicebio, GB155273, 1:10000).

### Preparation of CYEE and identification of its major chemical constituents

Pulverize dried *Corydalis yanhusuo* tubers were purchased from Jiangshan Wanli Chinese Materia Medica Co., Ltd. (Batch No.: Y2411029) Ethanol extraction of the Corydalis slices was carried out in accordance with the method provided in the 2020 edition of the Chinese Pharmacopoeia: A sample of 400 g was accurately weighed and placed in a 250–300 ml conical flask. 100 ml of 60 % ethanol was accurately added, the flask was tightly sealed, and cold maceration was carried out. The mixture was shaken frequently for the first 6 hours, then left to stand for a further 18 hours. It was rapidly filtered through a dry filter, and the extract was freeze-dried to yield powdered CYEE, with a yield of 1.85 % (w/w). Store the final product at −20 °C and reconstitute it in sterile phosphate-buffered saline (PBS) or the appropriate culture medium prior to use.

Chromatographic separation was performed using an Agilent SB C18 column (100 × 2.1 mm, 3.5 μm), with a mobile phase consisting of an aqueous solution containing 0.1 % formic acid and acetonitrile.

### Quantitative determination of dehydrocorydaline (DHC) in CYEE

To ensure the batch-to-batch consistency of CYEE, DHC was selected as the representative quality marker for quantitative analysis. DHC was quantified by LC-MS using an external standard method. Chromatographic separation was performed on a Waters XBridge BEH C18 column (4.6 × 150 mm, 3.5 μm) with 0.1 % formic acid aqueous solution and acetonitrile as the mobile phases under gradient elution. DHC standard solutions were used to establish the calibration curve, and the DHC content in CYEE was calculated according to the corresponding peak area and expressed as mg/g extract.

### Gas-shearing microfluidic equipment

The gas-shearing microfluidic platform primarily consists of four core modules: (1) a syringe pump for the precise and continuous delivery of the sodium alginate solution; (2) a nitrogen cylinder equipped with a flow meter for stable regulation of the gas flow rate; (3) a coaxial needle assembly, featuring a 27 G inner needle nested within an 18 G outer needle, with a 14 G needle fixed to the side wall of the outer needle as a gas inlet to achieve coaxial shearing of the liquid and gas phases; and (4) a collection bath for the recovery and solidification of the microspheres. Seal all connections within the device using pressure-resistant tubing to ensure the stability and reproducibility of the system.

### Preparation of sodium alginate microspheres

Load the SA precursor solution into a syringe pump and extrude it slowly through the inner tube of the coaxial needle at a constant flow rate. Introduce nitrogen gas through the annular gap between the inner and outer needles simultaneously. Form uniform droplets from the liquid stream via gas shearing. Collect the droplets directly in the CaCl₂ solution to induce instantaneous Ca²⁺-mediated ionic crosslinking and form structurally stable sodium alginate microspheres. Collect the microspheres from the collection bath via sedimentation, remove the supernatant, and wash the spheres three times with PBS.

### Characterization of sodium alginate microsphere structure and performance

Scanning electron microscopy (SEM) characterization: Wash the prepared microspheres three times with PBS, freeze at − 80 °C for 12 h, and lyophilize in a freeze dryer for 48 h. Observe and image the overall morphology and surface structure of the microspheres using SEM at various magnifications.

Mechanical characterization: Perform uniaxial compression testing at room temperature by placing freshly prepared microsphere samples between the parallel plates of a texture analyzer. Apply loading at a constant strain rate during the test, record stress-strain curves, and calculate the Young’s modulus.

Swelling experiment: Weigh the initial dry weight (W₀) of the lyophilized microspheres and incubate them statically in PBS (pH 7.4) at 37 °C. Remove the microspheres at predetermined time points (1, 6, 12, 24, and 48 h), gently blot excess surface liquid, and weigh the wet weight (Wt).

Degradation experiment: Evaluate the *in vitro* degradation behavior of the microspheres under simulated physiological conditions. Place the prepared sodium alginate microspheres in centrifuge tubes containing PBS (pH 7.4) and incubate with shaking at 37 °C. Collect samples at designated time points (1, 7, 14, 21, and 28 d), wash and lyophilize them, and weigh the residual dry weight (Wt).

### Cell culture

Culture human umbilical vein endothelial cells (HUVECs) and rat cardiomyocytes (H9C2) in complete DMEM supplemented with 10 % fetal bovine serum (FBS) and 1 % penicillin-streptomycin. Culture MSCs in complete MEM supplemented with 10 % FBS and 1 % penicillin-streptomycin. Incubate all cells at 37 °C in a humidified atmosphere containing 5 % CO₂. At 80–90 % confluence, discard the culture medium, wash the cells once with PBS, and digest with 0.25 % trypsin-EDTA. Collect the cells after terminating the reaction and centrifuge at 1000 rpm for 3 min. Discard the supernatant, resuspend the cells, and subculture them at a 1:2 ratio for subsequent experiments.

### Construction of CYEE and MSCs co-delivery carrier

Weigh an appropriate amount of CYEE lyophilized powder and add it to the pre-prepared sodium alginate (SA) precursor solution. Stir the mixture magnetically at room temperature until fully dispersed and dissolved. If dissolution is incomplete, apply brief ultrasonication to facilitate uniform drug dispersion to obtain a homogeneous CYEE-containing precursor solution. Subsequently, digest, centrifuge, and count MSCs in the logarithmic growth phase. Adjust the cell concentration to 1 × 10⁶ cells/mL using serum-free medium. Slowly add the cell suspension to the CYEE-containing SA precursor solution and mix thoroughly by gentle pipetting, avoiding vigorous stirring to prevent mechanical damage to the cells, thereby obtaining a composite precursor system co-loaded with CYEE and MSCs. Fabricate sodium alginate microspheres co-delivering CYEE and MSCs using gas-assisted microfluidic technology to achieve synergistic encapsulation and spatial co-localization of the drug and cells. Prepare microspheres delivering CYEE alone or MSCs alone by adding only CYEE or MSCs to the SA precursor solution following the same preparation protocol.

### Scratch experiment

Seed HUVECs in 6-well plates at a density of 2 × 10⁵ cells/well. Once the cell monolayer reaches full confluence, create uniform vertical scratches across the center of the wells using 200 μL sterile pipette tips. Remove detached cells and wash twice gently with PBS. Replace the medium with serum-free medium and apply the experimental treatments. Divide the cells into the following groups: Control (Con), CYEE + Gel, MSCs + Gel, and CYEE + MSCs + Gel. Continue incubation at 37 °C and 5 % CO₂, and capture images of the scratches at fixed fields of view at 0, 12, and 24 h. Measure the changes in scratch width using ImageJ software and calculate the cell migration rate.

### Tube formation experiment

Add pre-chilled Matrigel to 24-well plates (50 μL/well) and incubate at 37 °C for 30 min to allow complete gelation. Resuspend HUVECs in serum-free medium, adjust the cell density to 1 × 10⁴ cells/well, and seed them onto the Matrigel surface. Treat the cells according to the designated groups: Control (Con), CYEE + Gel, MSCs + Gel, and CYEE + MSCs + Gel. After incubation at 37 °C and 5 % CO₂ for 3–4 h, capture images from multiple randomly selected fields using a microscope. Quantify the total tube length and number of branch points using ImageJ.

### Animal experiments

Male Sprague-Dawley (SD) rats (200 ± 20 g) of SPF grade, purchased from Beijing Weitong Lihua Laboratory Animal Technology Co., Ltd., were used in this study. All animals were housed in standard SPF conditions with ad libitum access to food and water, and were acclimatised for at least 7 days prior to the start of the experiment. All animal experiments were conducted in accordance with relevant guidelines on the management and use of laboratory animals and were approved by the Laboratory Animal Ethics Committee of Guangzhou Medical University (Ethics Approval No.: GY2024-847).

A rat myocardial infarction model was established by permanent ligation of the left anterior descending artery (LAD). Prior to surgery, animals were anaesthetised with inhaled isoflurane at an induction concentration of 3–5 % and a maintenance concentration of 1–3 %; surgical procedures were performed once the animals had reached an appropriate depth of anaesthesia. The heart was then exposed via a left fourth intercostal thoracotomy, and the LAD was permanently ligated approximately 2–3 mm below the left atrial appendage. Rapid whitening of the distal myocardium following ligation was taken as an indication that the model had been successfully established. The sham surgery group underwent thoracotomy and cardiac exposure only, without ligation of the LAD.

All surgical procedures were performed under aseptic conditions. Post-operatively, the animals were placed in a temperature-controlled environment to recover, and their body weight, activity levels, food and water intake, and wound healing were monitored daily. No routine analgesics or prophylactic anti-infective treatment were administered during the experiment, and no significant infections or other serious post-operative complications were observed.

### Statistical analysis

All data are expressed as mean ± standard deviation (SD). Prior to conducting statistical analysis, a normality test is first performed on all data; for data that conform to a normal distribution, a test for homogeneity of variances is then carried out. Comparisons between two groups were performed using the two-tailed unpaired Student’s t-test, while comparisons among multiple groups were analyzed via one-way analysis of variance (ANOVA) followed by the Bonferroni correction. Statistical significance was set at *P* < 0.05, *P* < 0.01, *P* < 0.001, and* P* < 0.0001.

## Results

### Identification of the major chemical constituents of CYEE

As shown in the base chromatogram (Supplementary Fig. 1 A), under the established chromatographic conditions, the major components in CYEE achieved good baseline separation within 8 minutes; in conjunction with mass spectrometry data, six major alkaloid components were successfully identified, which, based on retention time, were tetrahydroparmatin, tetrahydropalmaretin, corydaline A, parmatin, berberine and dehydrocorydaline. Furthermore, analysis of the three-dimensional ion chromatogram (Supplementary Fig. 1B) reveals that each target peak exhibits an excellent signal-to-noise ratio in the corresponding mass channel, with no significant interference from endogenous matrix impurities, and their retention times correspond precisely with those in the total peak chromatogram.

### Quantitative analysis of DHC in CYEE

To further ensure the batch-to-batch consistency and reproducibility of the extract, DHC was selected as the representative quality marker for quantitative analysis. A satisfactory linear relationship between DHC concentration and peak area was obtained within the tested concentration range (Supplementary Table 1), with the calibration curve described by the equation *Y* = 1.1104e + 008 + 1.50461e + 006*X (*R²* = 0.9970). Based on the external standard calibration curve, the DHC content in CYEE was determined to be 0.169 mg/g extract. These results indicate that the prepared CYEE exhibited good batch consistency and can be reliably used for subsequent biological experiments.

### Preparation and characterization of sodium alginate microspheres

**Fig. 1:**
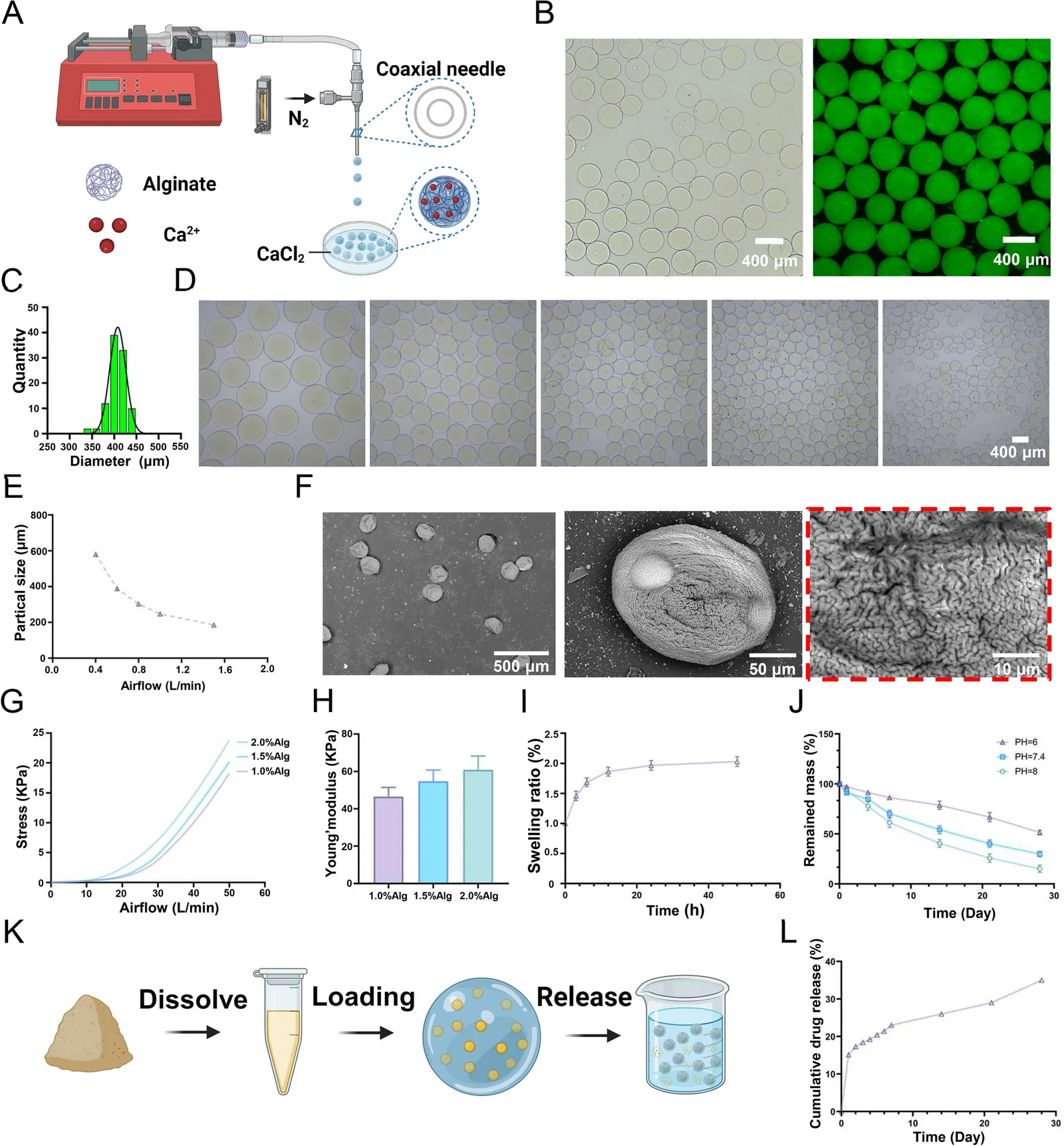
Characterization of the Morphology, Structure, and Physicochemical Properties of Sodium Alginate Microspheres, and the Preparation and Release Behavior of CYEE. **A** Schematic of microsphere preparation via gas-shearing. **B** Bright-field and fluorescence images of homogeneous sodium alginate microspheres. **C** Particle size distribution analysis of sodium alginate microspheres. **D** Bright-field images of microspheres of different sizes. **E** Relationship between airflow velocity and sodium alginate microspheres across different size ranges. **F** Scanning electron microscopy (SEM) images of sodium alginate microspheres. **G-J** Mechanical and physicochemical property tests of sodium alginate microspheres, including stress–strain curves (**G**), Young’s modulus analysis (**H**), swelling behavior (**I**), and in vitro degradation behavior (**J**). **K** Schematic of CYEE release. **L** In vitro release curve of CYEE from sodium alginate microspheres. Statistical data are expressed as mean ± SD (n = 3)

This strategy utilizes gas as the shearing phase and eliminates the need for an oil phase or surfactants, minimizing the potential negative impacts of organic solvents and surfactants on drug stability and cell viability, which is common in traditional emulsion methods. Sodium alginate (SA) microspheres with highly uniform morphology and structure were successfully fabricated using gas-assisted microfluidic technology (Fig. 1A). Bright-field microscopy showed that the resulting microspheres were regularly spherical, with well-defined boundaries and a uniform distribution. Fluorescence imaging further confirmed a homogeneous fluorescence signal throughout the microspheres (Fig. 1B). Particle size analysis showed that the microspheres had a mean diameter of 408.01 ± 18.17 μm, with a unimodal size distribution and a coefficient of variation (CV) of 4.45 % (Fig. 1C).

As shown in Fig. 1D, under otherwise identical fabrication conditions, the diameter of the SA microspheres gradually decreased with increasing gas flow velocity. Quantitative analysis of microsphere size (Fig. 1E) further confirmed an inverse relationship between gas flow velocity and microsphere diameter, demonstrating that microsphere size could be effectively regulated by adjusting the gas flow velocity. SEM images revealed a generally smooth microsphere surface with uniformly distributed microporous structures (Fig. 1F).

The mechanical properties of microspheres prepared with different SA concentrations (1.0 %, 1.5 %, and 2.0 %) were subsequently evaluated. The stress sustained by the microspheres at the same strain increased with increasing SA concentration, indicating enhanced mechanical properties (Fig. 1G). The Young's modulus of the 2.0 % SA microspheres was 60.9 ± 7.3 kPa (Fig. 1H). Considering their mechanical properties, structural stability, and suitability for in vivo application, 2.0 % SA microspheres were selected as the standard material system for all subsequent experiments. Swelling experiments showed that the microspheres reached a swelling ratio of approximately 200.0 % after 48 h under physiological conditions (Fig. 1I). In vitro degradation experiments demonstrated gradual degradation of the microspheres in a pH 7.4 environment, with approximately 30.0 % of the original mass remaining after 28 d (Fig. 1J). Fig. 1K illustrates the preparation of CYEE and its subsequent loading into SA microspheres for in vitro release studies. Release analysis showed sustained release of CYEE from the SA microspheres, with an initial burst release followed by a gradual stabilization of the release rate, reaching a cumulative release of 35.30 % at day 28 (Fig. 1L).

### Biocompatibility evaluation of the sodium alginate microsphere delivery system

**Fig. 2:**
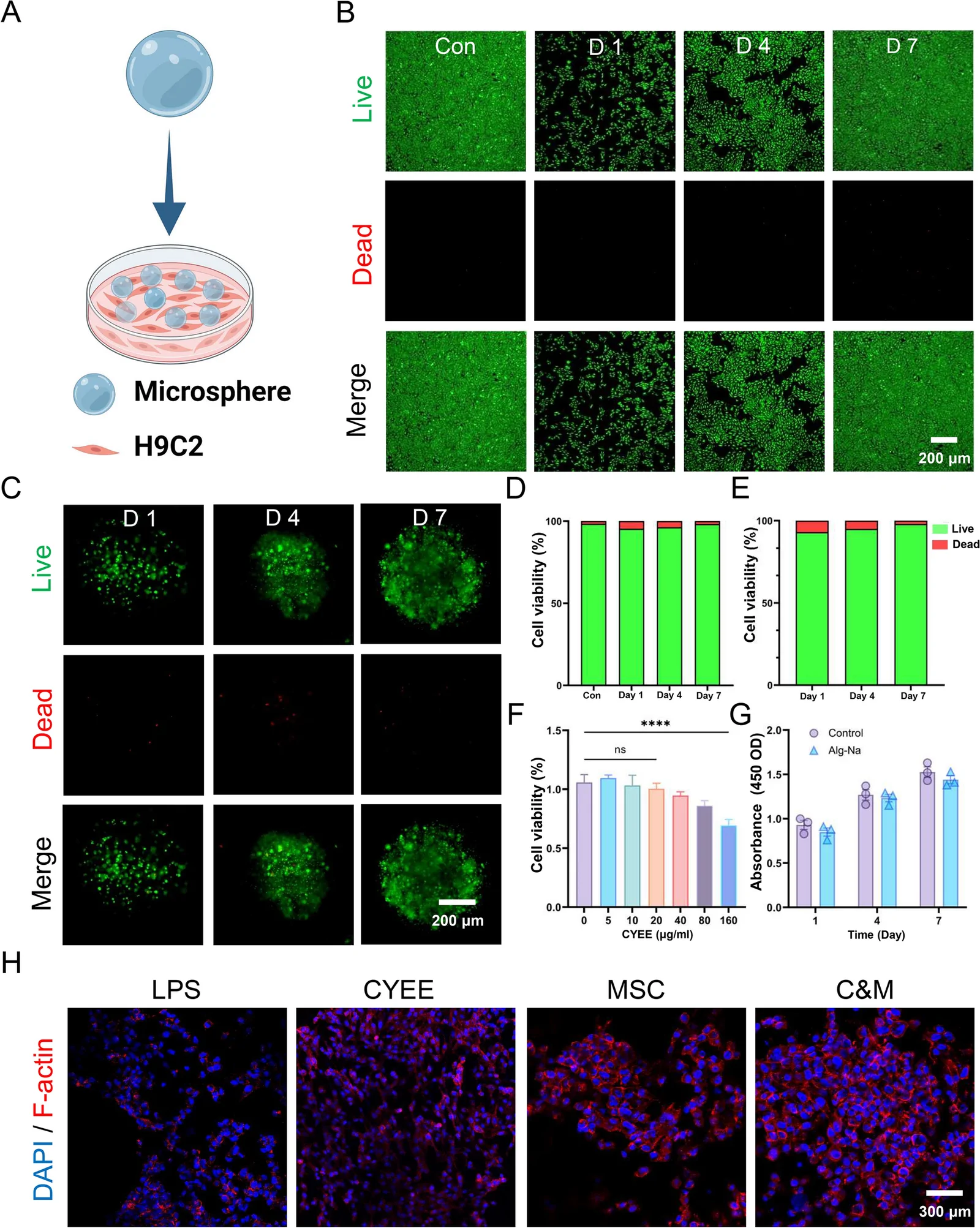
Biocompatibility Evaluation of the Sodium Alginate Microsphere Co-loading System. **A** Schematic diagram of sodium alginate microspheres co-cultured with H9C2 cells. **B** Live/dead staining images of H9C2 cardiomyocytes co-cultured with sodium alginate microspheres for 1, 4, and 7 d; the control group (Con) had no microspheres. **C** Live/dead staining images of CYEE and MSCs co-loaded within sodium alginate microspheres. **D** Quantitative statistical analysis of the corresponding live/dead staining results. **E** Quantitative analysis of MSCs viability in the co-loading system. **F** CCK-8 cell viability assay results for H9C2 cells treated with different concentrations of CYEE (0, 10, 20, 40, 80, and 160 μg/mL). **G** Cell proliferation capacity (CCK-8) of the co-loaded MSCs. **H** F-actin staining images of MSCs under different treatment conditions (Con, LPS, LPS + CYEE, LPS + MSCs, LPS + MSCs + CYEE). Statistical data are expressed as mean ± SD (n = 3). Statistical analysis was performed using one-way ANOVA; significant differences are marked as ****p < 0.001

To evaluate the biological safety and cytocompatibility of the proposed delivery system, a series of in vitro experiments were performed. The overall design of the co-culture strategy between sodium alginate microspheres and cells is illustrated in Fig. 2A. Live/dead staining showed that H9C2 cardiomyocytes maintained high viability following co-culture with blank sodium alginate microspheres (Fig. 2B), with cell viability remaining above 90 % at all time points and showing no significant difference from the control group (Fig. 2C). These results indicate good cytocompatibility of the sodium alginate microsphere carrier. The cytotoxicity of CYEE was further evaluated in H9C2 cells. Cell viability remained above 90 % at CYEE concentrations of 10–40 μg/mL, whereas significant inhibition of cell proliferation was observed at 160 μg/mL (Fig. 2D), indicating good cellular tolerance to CYEE within an appropriate concentration range.

The cytocompatibility of MSCs co-loaded with CYEE in sodium alginate microspheres was subsequently evaluated. Live/dead staining and quantitative analysis showed that MSCs viability remained above 90 % following encapsulation (Fig. 2E, F). Moreover, CCK-8 analysis demonstrated a progressive increase in MSCs proliferation over the 7-day culture period (Fig. 2G), indicating that the co-delivery system maintained both MSCs viability and proliferative capacity.

To further evaluate the protective effects of the different treatments under inflammatory conditions, F-actin staining was performed in endothelial cells (Fig. 2H). LPS stimulation resulted in marked disruption of F-actin organization, whereas CYEE or MSCs treatment partially restored cytoskeletal integrity. Notably, the combined treatment group exhibited the most pronounced improvement in F-actin organization, with enhanced fiber continuity and cell spreading, suggesting a synergistic protective effect of CYEE and MSCs under inflammatory conditions.

### Promotion of cell growth, migration, and inflammation tolerance by the sodium alginate microsphere delivery system

Angiogenesis is essential for myocardial repair following MI by improving oxygen and nutrient supply to the injured tissue. In this study, scratch and tube formation assays were performed to evaluate the effects of the delivery system on endothelial cell migration and angiogenic activity.

**Fig. 3:**
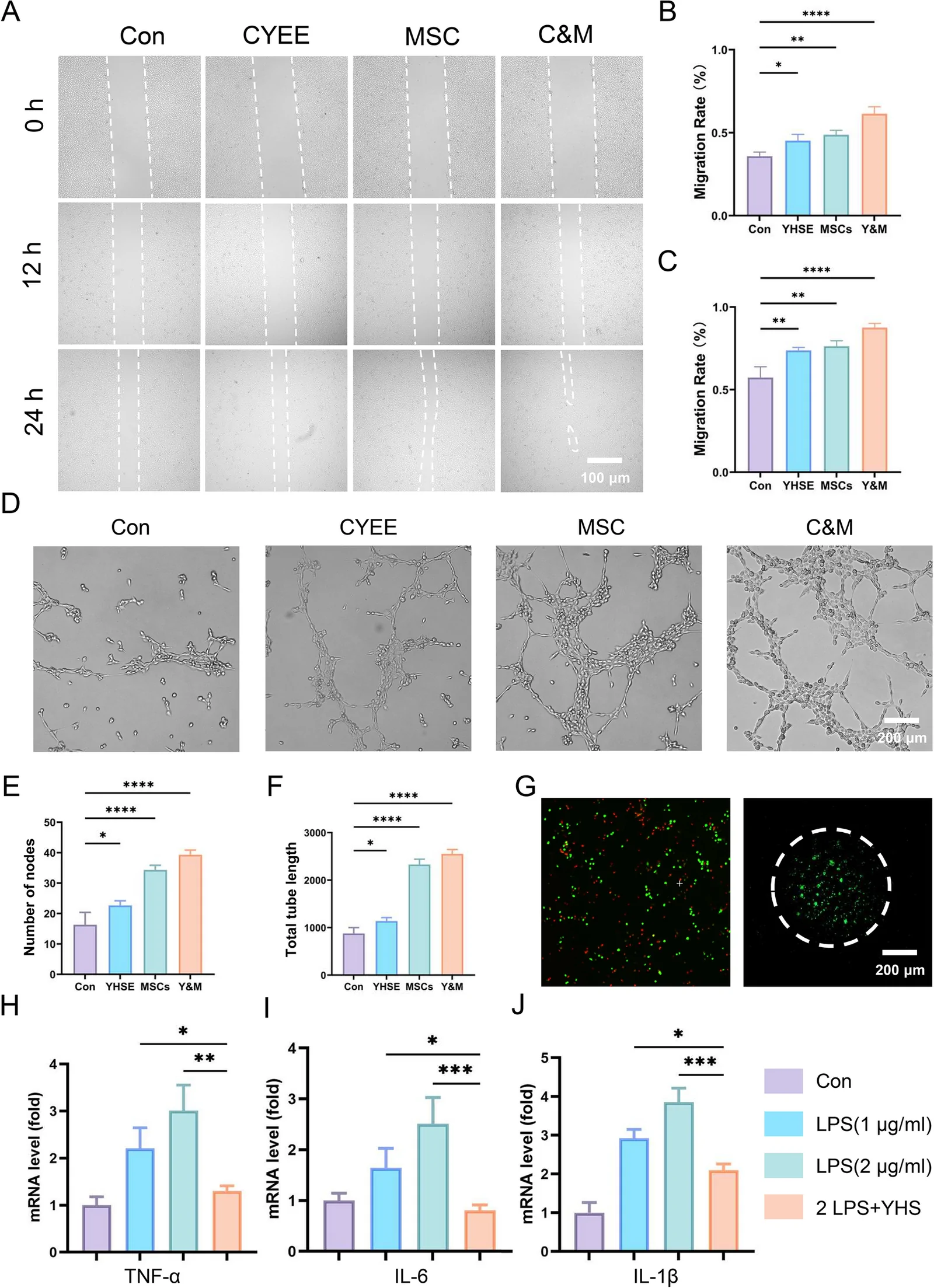
Promotion of in Vitro Migration and Angiogenesis by the Sodium Alginate Delivery System. **A** Representative images of the endothelial cell scratch healing assay at 0, 12, and 24 h for different treatment groups. **B** Quantitative analysis of cell migration rate at 12 h. **C** Quantitative analysis of cell migration rate at 24 h. **D** Representative fluorescence images of the endothelial cell tube formation assay. **E** Quantitative statistical analysis of the number of nodes in the tube formation assay. **F** Quantitative statistical analysis of the total tube length in the tube formation assay. **G** Live/dead staining images for the evaluation of MSCs inflammation tolerance under LPS-induced inflammatory conditions. **H-J** Detection of TNF-α, IL-6, and IL-1β expression levels in cardiomyocytes under different concentrations of LPS induction in the in vitro anti-inflammatory capacity evaluation. Statistical data are expressed as mean ± SD (n = 3). Statistical analysis was performed using one-way ANOVA; significant differences are marked as *p < 0.05, **p < 0.01, and ***p < 0.001, ****p < 0.0001

Scratch assays showed that both CYEE and MSCs treatment promoted HUVECs migration, whereas the CYEE + MSCs + Gel group exhibited the most pronounced effect, with nearly complete wound closure at 24 h (Fig. 3A). Quantitative analysis at 12 and 24 h further confirmed the enhanced migratory capacity of the combined treatment group (Fig. 3B, C). Similarly, tube formation assays demonstrated enhanced angiogenic activity following treatment with CYEE- or MSCs-loaded microspheres (Fig. 3D). Notably, the CYEE + MSCs + Gel group formed the most extensive and interconnected tubular network, with significantly increased numbers of nodes and total tube length compared with the control and monotherapy groups (Fig. 3E, F), indicating a synergistic pro-angiogenic effect of CYEE and MSCs.

The protective effects of the microsphere delivery system on MSCs under inflammatory conditions were further evaluated using an LPS-induced inflammatory model. LPS stimulation markedly reduced MSCs viability, whereas encapsulated MSCs maintained higher viability under the same conditions (Fig. 3G), indicating that the microsphere system improved MSCs tolerance to inflammatory stress.

The anti-inflammatory activity of CYEE was subsequently evaluated in LPS-stimulated H9C2 cells. LPS treatment increased the expression of TNF-α, IL-6, and IL-1β, whereas CYEE treatment significantly reduced the expression of these inflammatory cytokines under 2 ng/mL LPS stimulation (Fig. 3I, J). These results indicate that CYEE effectively attenuated the LPS-induced inflammatory response.

### In vivo efficacy and safety evaluation of the sodium alginate microsphere delivery system

**Fig. 4:**
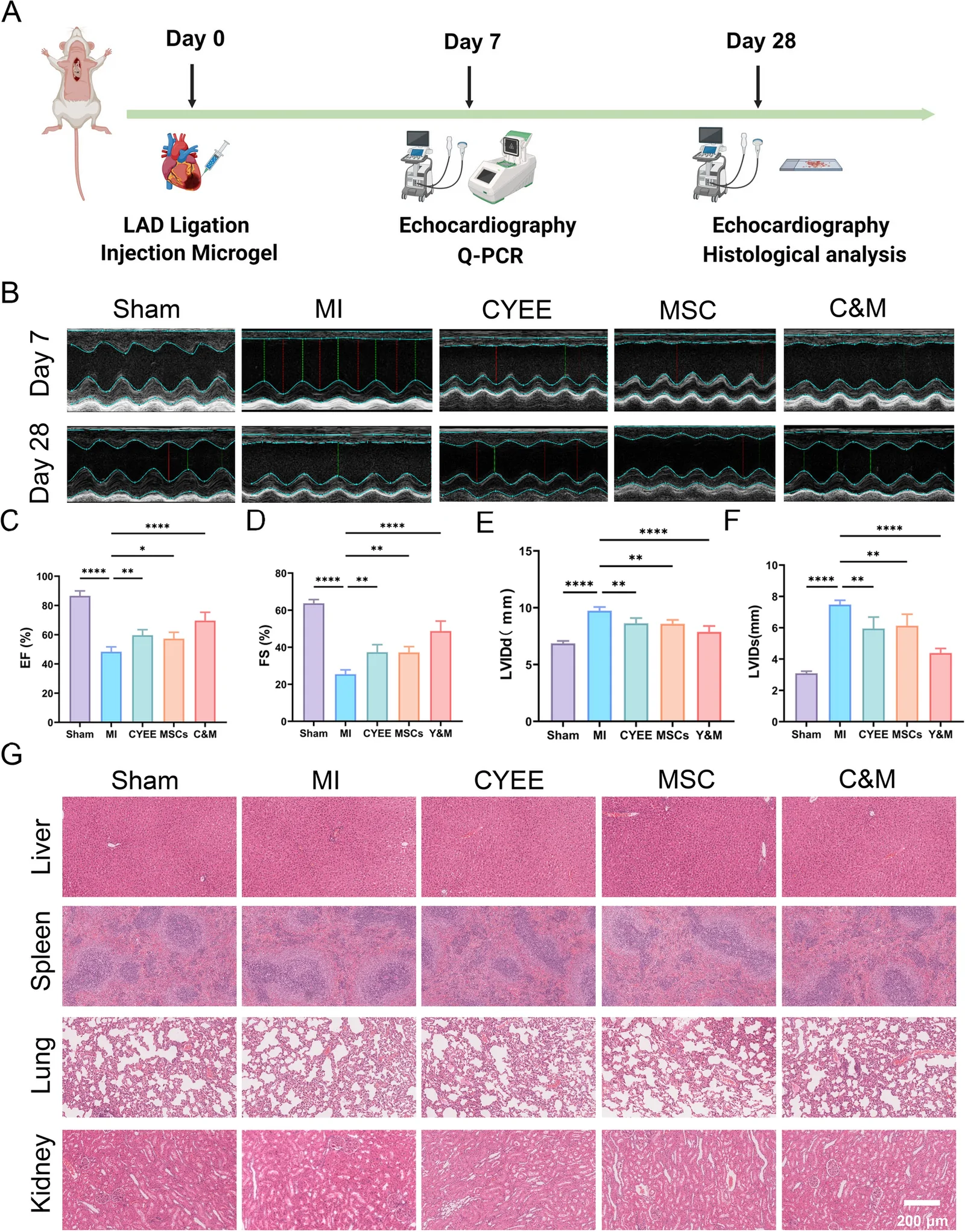
Evaluation of in Vivo Therapeutic Efficacy in a Rat Myocardial Infarction Model. **A** Schematic diagram showing the experimental workflow for myocardial infarction model construction, therapeutic intervention, echocardiographic evaluation, and tissue sampling. **B** Representative echocardiographic images of the Control group (Sham), myocardial infarction group (MI), CYEE + Gel group, MSCs + Gel group, and CYEE + MSCs + Gel group at 7 and 28 d after the surgery. **C-F** Quantitative statistical analysis of EF, FS, LVIDd, and LVIDs. **G** H&E staining images of major organs (liver, spleen, lung, and kidney). Statistical data are expressed as mean ± SD (n = 4). Statistical analysis was performed using one-way ANOVA; significant differences are marked as *p < 0.05, **p < 0.01, and ***p < 0.001, ****p < 0.0001

To further verify the therapeutic efficacy and biosafety of the sodium alginate microsphere delivery system *in vivo*, a rat MI model was successfully established via LAD ligation. The experimental groups included the Sham group, MI group, CYEE + Gel group, MSCs + Gel group, and CYEE + MSCs + Gel group. Cardiac function was evaluated by echocardiography on days 7 and 28 after surgery, followed by histological analysis (Fig. 4A).

Echocardiographic analysis showed marked cardiac dysfunction in the MI group, as evidenced by reduced Ejection Fraction (EF) and Fractional Shortening (FS) and increased Left-ventricular internal dimension at end-diastole (LVIDd) and Left-ventricular internal dimension at end-systole (LVIDs) compared with the Sham group (Fig. 4B). Both CYEE + Gel and MSCs + Gel treatment partially improved cardiac function. Notably, the CYEE + MSCs + Gel group exhibited the most pronounced therapeutic effect, with significantly increased EF and FS and reduced LVIDd and LVIDs compared with the MI and monotherapy groups (Fig. 4C–F). These results demonstrate that the combined treatment more effectively attenuated post-MI ventricular remodeling and improved cardiac function than either monotherapy.

The systemic biosafety of the delivery system was further evaluated by H&E staining of major organs at the experimental endpoint. No obvious histopathological abnormalities were observed in the liver, spleen, lung, or kidney of the treatment groups compared with the Sham group (Fig. 4G), indicating good in vivo biosafety of the sodium alginate microsphere-based delivery system.

### Histological and immunofluorescence analysis reveals improved myocardial structural repair and remodeling

**Fig. 5:**
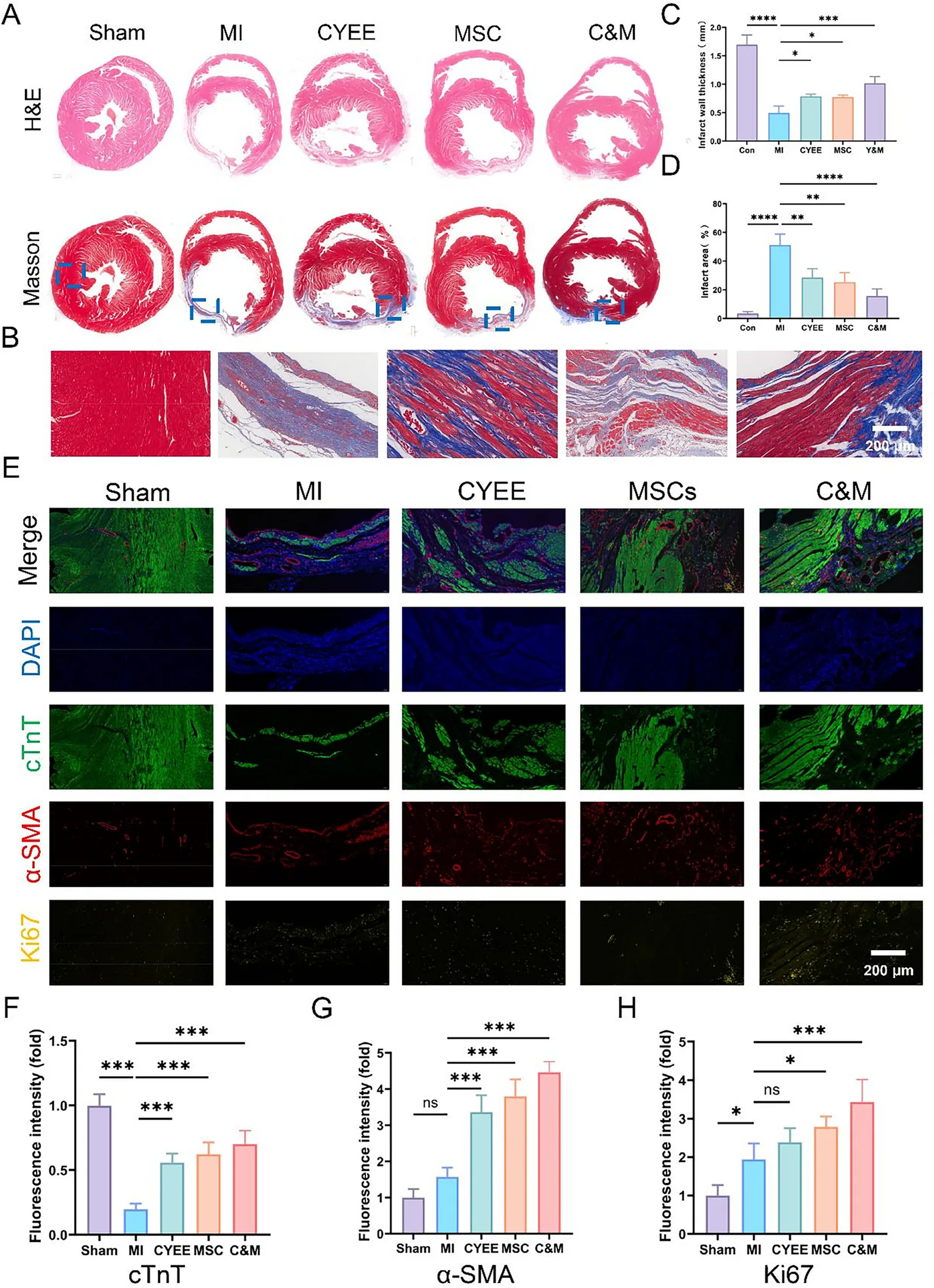
Histological and Immunofluorescence Analysis of Myocardial Repair and Remodeling. **A** H&E and Masson’s staining images of heart tissue sections from different treatment groups. **B** Magnified images of the infarcted myocardial area in Masson’s staining. **C** Quantitative analysis of left ventricular wall thickness in the infarcted area. **D** Quantitative analysis of the infarct area percentage. **E** Representative immunofluorescence staining images of heart tissue showing the expression of cTnT, α-SMA, and Ki67, and their merged images with DAPI. **F–H** Quantitative statistical analysis of the fluorescence intensities of cTnT, α-SMA, and Ki67. Statistical data are expressed as mean ± SD (n = 4). Statistical analysis was performed using one-way ANOVA; significant differences are marked as *p < 0.05, **p < 0.01, and ***p < 0.001, ****p < 0.0001

To evaluate the long-term effects of the treatment on post-MI cardiac remodeling, histological analyses were performed. H&E and Masson’s trichrome staining showed severe myocardial structural disruption and extensive collagen deposition in the MI group (Fig. 5A). Both CYEE + Gel and MSCs + Gel partially improved myocardial structure and reduced fibrosis. Notably, the CYEE + MSCs + Gel group exhibited the most pronounced structural recovery, with improved myocardial organization and markedly reduced collagen deposition. Enlarged images of the infarct region further confirmed reduced scar formation in the combined treatment group (Fig. 5B).

Quantitative analysis further supported these observations. Compared with the MI group, infarct wall thickness increased and infarct size decreased in the treatment groups, with the most pronounced improvements observed in the CYEE + MSCs + Gel group (Fig. 5C-D). These findings indicate that the combined treatment effectively attenuated post-MI myocardial remodeling and fibrosis.

Immunofluorescence staining was subsequently performed to evaluate myocardial repair and proliferation-associated markers. Compared with the Sham group, MI group, α-SMA, cTnT, and Ki67 signals were increased following treatment, with the CYEE + MSCs + Gel group showing the most pronounced enhancement (Fig. 5E). Quantitative analysis further confirmed significantly increased fluorescence intensities of these markers in the combined treatment group compared with the MI and monotherapy groups (Fig. 5F–H), suggesting enhanced myocardial structural reconstruction and proliferation-associated activity.

To further evaluate changes in the inflammatory microenvironment, myocardial tissues were stained for CD86 and CD206, markers associated with pro-inflammatory and reparative macrophage phenotypes, respectively. Compared with the MI group, the CYEE + MSCs + Gel group exhibited reduced CD86-positive signals and increased CD206-positive signals (Supplementary Fig. 2), indicating a shift toward a reparative macrophage phenotype. These findings were consistent with the reduced expression of TNF-α, IL-6, and IL-1β and the improved cardiac function observed following combined treatment.

## Discussion

The progressive decline of cardiac function after acute myocardial infarction is essentially rooted not only in the irreversible and massive loss of cardiomyocytes but also in the persistent inflammatory imbalance and pathological microenvironment within the infarcted region. Although revascularization strategies such as PCI and CABG have significantly reduced acute-phase mortality and limited the infarct area to some extent, these strategies primarily target blood flow restoration itself. They struggle to reverse established cardiomyocyte apoptosis and necrosis and cannot effectively block the subsequent inflammatory cascade and pathological ventricular remodeling processes. Therefore, achieving sustained and stage-appropriate microenvironmental regulation within the infarcted area has become a critical scientific challenge limiting the efficacy of myocardial repair.

The inflammatory response following myocardial infarction exhibits distinct spatio-temporal heterogeneity. During the acute phase, moderate inflammation facilitates the clearance of necrotic tissue and the initiation of early repair. However, persistent or excessive activation of the inflammatory response exacerbates oxidative stress injury, induces cardiomyocyte apoptosis, and promotes abnormal fibroblast activation, ultimately leading to progressive ventricular remodeling and continuous deterioration of cardiac function. Conventional drugs typically target single or limited pathways and rely on passive diffusion for therapeutic effect, making it difficult to maintain stable, stage-appropriate effective concentrations within the infarcted region, which has significantly restricted their therapeutic potential during the myocardial repair phase. The results of this study demonstrate that CYEE has effectively alleviated infarction-related inflammatory responses both *in vitro* and *in vivo* by significantly downregulating the expression of key inflammatory cytokines such as TNF-α, IL-6, and IL-1β, thereby exerting initial therapeutic effects and creating relatively mild microenvironmental conditions for subsequent tissue repair. This finding has not only expanded the application boundaries of the traditional pharmacological roles of *Corydalis yanhusuo,* but also provided a basis for the participation of active components of traditional Chinese medicine in myocardial repair.

Notably, in this study, CYEE did not act as a short-acting anti-inflammatory intervention but achieved local enrichment and sustained release through the sodium alginate microsphere delivery system. This strategy has effectively avoided the limitations of traditional administration (such as high peaks and short maintenance times), ensuring that the anti-inflammatory effect matches the temporal scale of the myocardial repair process. This characteristic is particularly crucial for the smooth transition from the inflammatory phase to the repair phase after MI, and has provided a mechanistic explanation for the long-term efficacy observed in cardiac function recovery and structural improvement in animal models.

LC-MS analysis revealed that CYEE primarily contains alkaloids such as tetrahydropalmatine, berberine, palmatine and dehydrocorydaline. Previous studies have shown that tetrahydropalmatine can inhibit hypoxia-induced cardiomyocyte apoptosis and inflammatory responses by activating the PI3K/Akt signalling pathway [38]; berberine possesses well-established anti-inflammatory, antioxidant and cardioprotective effects, and can improve cardiac function and alleviate ventricular remodelling following myocardial infarction by regulating signalling pathways such as Akt, p38 MAPK and SIRT1 [39]; palmatine exerts anti-inflammatory and anti-fibrotic effects by activating the AMPK/Nrf2 signalling pathway, inhibiting STAT3-mediated fibroblast activation and regulating the inflammatory phenotype of macrophages [40]; Dehydrocorydaline, meanwhile, is considered one of the primary contributors to the anti-inflammatory activity of Corydalis, as it can significantly reduce the expression of pro-inflammatory factors such as TNF-α and IL-6 by inhibiting the TRAF6/NF-κB signalling pathway [41]. Furthermore, existing research has confirmed that Corydalis extracts can alleviate myocardial ischaemia-reperfusion injury and improve cardiac function. It is worth noting that, in recent years, a growing body of evidence has indicated that the active alkaloids in Corydalis also possess the ability to regulate macrophage phenotypic conversion. Among these, dehydrocorydaline promotes M2-type polarisation by inhibiting the JAK1/STAT3 signalling pathway, whilst tetrahydroparmatin suppresses M1-type macrophage activation and promotes M2-type polarisation by regulating inflammation-related signalling pathways such as PI3K/Akt and TLR4/NF-κB, thereby alleviating tissue inflammatory damage and promoting repair [20, 42, 43]. Consistent with the aforementioned reports, the supplementary CD86/CD206 immunofluorescence results from this study showed that CD86-positive cells were abundantly expressed in the myocardial tissue of the MI group, whilst CD206 expression was low; in contrast, CD86 expression was markedly reduced in the CYEE + MSCs combination therapy group, whilst CD206-positive cells increased significantly, suggesting that the combination therapy promoted the shift of macrophages from a pro-inflammatory phenotype to a reparative phenotype. Taking into account the significant reduction in TNF-α, IL-6 and IL-1β levels, as well as the improvement in cardiac function, we hypothesise that the cardioprotective effects of CYEE may stem from anti-inflammatory, anti-oxidative stress and anti-apoptotic effects synergistically mediated by multiple alkaloids. These effects may reshape the post-infarction inflammatory microenvironment by regulating macrophage polarisation, thereby providing more favourable conditions for MSCs survival, the exertion of their paracrine functions and subsequent tissue repair.

On this basis, this study has further elucidated the critical role of microenvironmental improvement in maximizing the effects of cell therapy. Numerous studies have shown that the reparative role of MSCs in myocardial infarction primarily depends on paracrine mechanisms, yet their long-term viability in the infarcted area is generally low. Our *in vitro* experiments have revealed that MSCs viability decreases significantly under inflammatory stimulation. However, CYEE-mediated microenvironmental regulation significantly improves the tolerance of MSCs to inflammatory stress and maintains their normal morphology and viability. *In vivo* results have indicated that the synergistic therapeutic effects of MSCs are fully unleashed following the sustained anti-inflammatory action of CYEE, manifested as significant improvements in cardiac function indices, increased infarct wall thickness, and marked attenuation of myocardial structural remodeling.

The sodium alginate microspheres fabricated via gas-assisted microfluidic technology have provided critical technical support for the construction of a synergistic myocardial repair strategy centered on microenvironmental regulation. This fabrication method requires no oil phase or surfactants, avoiding potential damage to drug and cell activity caused by residual organic solvents and creating a cell-friendly environment for the co-loading of CYEE and MSCs. The comprehensive advantages of these microspheres, including mechanical matching, controllable degradation behavior, and a porous network structure, not only facilitate the sustained release of CYEE to provide initial therapeutic intervention through anti-inflammatory and anti-oxidative stress regulation, but also provide effective physical protection for encapsulated MSCs, ensuring high viability and a stable functional state under the conditions of cardiac pulsation and inflammatory stress. Furthermore, their excellent injectability and tissue adaptability have provided practical feasibility for precise local delivery and dynamic microenvironmental regulation. Accordingly, this study has proposed a comprehensive therapeutic strategy that achieves myocardial repair through the synergy of a traditional Chinese medicine extract and MSCs. Through its anti-inflammatory and anti-oxidative stress biological effects, CYEE has provided initial therapeutic intervention in MI. By actively regulating the inflammatory microenvironment, it induces and amplifies the adaptive response of MSCs to environmental changes, thereby significantly enhancing their viability and paracrine function and promoting the synergistic amplification of cell therapy effects. Compared with monotherapy with drugs or cells alone, this strategy better aligns with the biological characteristics of the complex pathological processes following MI, demonstrating the advantages of multi-level and sequential regulation. The combination of CYEE-mediated microenvironmental remodeling and MSCs-mediated dynamic repair has provided a new comprehensive therapeutic model with potential translational value for the reconstruction of myocardial structure and function following infarction.

Although this study has demonstrated that the CYEE-MSCs synergistic delivery system can effectively improve the inflammatory microenvironment following myocardial infarction and promote myocardial repair, certain limitations remain. Firstly, this study focused primarily on a drug-cell synergistic delivery system based on sodium alginate microspheres, with the emphasis on verifying its overall therapeutic efficacy and the feasibility of improving the inflammatory microenvironment and enhancing the therapeutic effects of MSCs; consequently, no systematic control groups using blank sodium alginate microsphere, free CYEE or free MSCs were established, nor were further studies conducted on the kinetics of local drug release. It is therefore not yet possible to comprehensively quantify the advantages of the microsphere carrier over free administration across multiple aspects, such as local drug retention, cell protection and delivery efficiency. Secondly, whilst this study preliminarily confirmed through CD86 and CD206 immunofluorescence staining that the combination therapy promotes the transition of macrophages from a pro-inflammatory phenotype to a reparative phenotype, systematic research into the specific molecular regulatory mechanisms and the dynamic changes in other immune cell subsets remains lacking. Further investigation is required, combining techniques such as flow cytometry, multiplex immunofluorescence, single-cell sequencing and multi-omics analysis to elucidate the mechanisms underlying CYEE-mediated remodelling of the immune microenvironment. Finally, due to the high perioperative mortality rate in the myocardial infarction model and constraints such as the experimental cycle, the sample size for the in vivo experiments in this study was relatively limited. Although all evaluation indicators showed consistent trends and reached statistical significance, it remains necessary to further validate the findings of this study in animal models with larger sample sizes and long-term follow-up to enhance the reliability and generalisability of the conclusions. In the future, we will establish a range of control systems involving free drugs, free cells and carrier-mediated delivery. By combining pharmacokinetic analysis, high-sensitivity in vivo cell tracing, multi-omics analysis and mechanistic studies, we aim to systematically elucidate the mechanisms underlying microsphere-mediated local sustained-release, microenvironment remodelling and tissue repair, thereby providing a more robust experimental basis for the clinical translation of this synergistic therapeutic strategy.

## Supplementary Information

Supplementary Material 1.

## Data Availability

All data related to this study can be obtained from the authors on reasonable request.

## Abbreviations

MI: Myocardial Infarction
CYEE: Corydalis yanhusuo ethanolic extract
CAD: Coronary artery disease
PCI: Percutaneous coronary intervention
CABG: Coronary artery bypass grafting
TCM: Traditional Chinese medicine
MSCs: Mesenchymal stem cells
SA: Sodium alginate
HUVECs: Human umbilical vein endothelial cells
LPS: Lipopolysaccharide
TNF-α: Tumor Necrosis Factor-alpha
IL-1β: Interleukin-1β
IL-6: Interleukin-6
cTnT: Cardiac troponin T
α-SMA: Alpha-smooth muscle actin
Ki67: Marker of Proliferation Ki-67
