# Supplementary material for: Treatment of myocardial infarction via sodium alginate microsphere-mediated co-delivery of Corydalis yanhusuo ethanolic extract and mesenchymal stem cells
Source: Chin Med. 2026 Oct 30;21:234. doi: 10.1186/s13020-026-01557-z (PMC13721202; doi:10.1186/s13020-026-01557-z)
Supplement: Supplementary file 1 [file 13020_2026_1557_MOESM1_ESM.docx]

**Supplemental Material**

**Treatment of Myocardial Infarction via Sodium Alginate Microsphere-mediated Co-delivery of Corydalis yanhusuo Ethanolic Extract and Mesenchymal Stem Cells**

**Authors**

Yuanye Dang^1, †^, Junjie Cai^1, †^, Yongxian Li^1, †^, Xudong Qin^1^, Jun Shen^1^, Lian Duan^3^, Qingyun Lu^1^, Xinyue Zhang^1^, Wenqi Xiao^1^, Ruoxuan Liu^4^, Guosheng Tang^1,^ *, Ailan Chen^2,^ *, Huaibin Wang^1,^ *

**Institutions**

^1^ Guangzhou Municipal and Guangdong Provincial Key Laboratory of Molecular Target & Clinical Pharmacology, the NMPA and State Key Laboratory of Respiratory Disease, School of Pharmaceutical Sciences, Guangzhou Medical University, Guangzhou 511436, China.

^2^ Department of Cardiology, The First Affiliated Hospital of Guangzhou Medical University, China State Key Laboratory for Respiratory Disease, Guangzhou, 510120, China.

^3^ Phase I Clinical Trial Center, The Second Affiliated Hospital, Guangzhou Medical University, No. 63, South Road of Aisa Games City, Panyu District, Guangzhou 511400, China.

^4^ Department of Pharmacy, The Affiliated Traditional Chinese Medicine Hospital, Guangzhou Medical University, Guangzhou, 510645, China.

^†^ Yuanye Dang, Junjie Cai and Yongxian Li have equally contributed to this article.

*** Corresponding author:**

Guosheng Tang (Email: [guoshengtang@gzhmu.edu.cn](mailto:guoshengtang@gzhmu.edu.cn)), Ailan Chen (Email: [chenailan1997@gzhmu.edu.cn](mailto:chenailan1997@gzhmu.edu.cn)) and Huaibin Wang (Email: [y3qgz10lhh@icloud.com](mailto:y3qgz10lhh@icloud.com))

Adress: Guangzhou Municipal and Guangdong Provincial Key Laboratory of Molecular Target & Clinical Pharmacology, the NMPA and State Key Laboratory of Respiratory Disease, School of Pharmaceutical Sciences, Guangzhou Medical University, Guangzhou 511436, China. Email: [guoshengtang@gzhmu.edu.cn](mailto:guoshengtang@gzhmu.edu.cn). Phone: +86-13057566046.

**Supplemental Figures**

Supplementary Figure 1. Identification of CYEE components.

**Supplementary Figure 1. Identification of CYEE components.** A. HPLC-HRMS chromatogram of CYEE. 1. Tetrahydroparmatin; 2. Tetrahydropalmaretin; 3. Corydalis A; 4. Parmatin; 5. Berberine; 6. Dehydrocorydalin. HPLC conditions: Agilent SB C18 column (100 × 2.1 mm, internal diameter = 3.5 μm). Mobile phase: Milli-Q water and acetonitrile containing 0.1% formic acid. B. Three-dimensional HPLC-HRMS chromatograms of YHSE. From top to bottom: chromatogram of the total alkaloid peak, dehydrocorydaline, berberine, berberine, corydaline A, tetrahydroberberine and tetrahydroparmatine. HPLC conditions: The column used was an Agilent SB C18 column (100 × 2.1 mm, internal diameter = 3.5 μm), with Milli-Q water (containing 0.1% formic acid) and acetonitrile as the mobile phase.

**Supplemental Table**

Table 1 Calibration curve data of dehydrocorydaline (DHC) standard solutions and quantitative analysis of DHC in CYEE.

| Filename | Area | Specified Amoun | Calculated Amoun | %Diff | %RSD-AMT | Units |
| --- | --- | --- | --- | --- | --- | --- |
| 100ppb | 231054257 | 100.000 | 79.764 | -20% | 0.0% | ppb |
| 200ppb | 429989672 | 200.000 | 211.982 | 6% | 0.0% | ppb |
| 400ppb | 721680558 | 400.000 | 405.847 | 1% | 0.0% | ppb |
| 800ppb | 1353228250 | 800.000 | 825.590 | 3% | 0.0% | ppb |
| 1000ppb | 1580764161 | 1000.000 | 976.816 | -2% | 0.0% | ppb |
| CYEE | 316441730 | NA | 136.515 | NA | NA | ppb |
